# Effect of metabolic uncoupler, 3,3′,4′,5-tetrachlorosalicylanilide (TCS) on *Bacillus subtilis*: biofilm formation, flocculability and surface characteristics†

**DOI:** 10.1039/c8ra02315h

**Published:** 2018-05-01

**Authors:** Xiao-Chi Feng, Wan-Qian Guo, He-Shan Zheng, Qing-Lian Wu, Hai-Chao Luo, Nan-Qi Ren

**Affiliations:** State Key Laboratory of Urban Water Resource and Environment, Harbin Institute of Technology 73 Huanghe Road Harbin Heilongjiang 150090 P. R. China guowanqian@126.com

## Abstract

In order to understand the inhibitory mechanism of metabolic uncoupler in biofilm, this study investigated the effect of TCS on *B. subtilis* biofilm formation, flocculability, surface characteristics and thermodynamic properties. An optimal concentration of TCS, a metabolic uncoupler, was observed to substantially inhibit biofilm formation and the secretion of extracellular polymeric substances (EPS). The effect of TCS on the zeta potential and flocculability of bacterial suspension implied the addition of 100 μg L^−1^ TCS increased the net negative charge of cell surface which induced the reduction of *B. subtilis* flocculability. Meanwhile, the effects of TCS on bacterial surfacial thermodynamic properties were analyzed by the Derjaguin–Landau–Verwey–Overbeek (DLVO) and extend DLVO (XDLVO) theories. As DLVO and XDLVO predicted, the primary energy barrier between bacterial cells incubated with 100 μg L^−1^ TCS were increased compared to that of control, indicating that *B. subtilis* incubated with 100 μg L^−1^ TCS must consume more energy to aggregate or form biofilm.

## Abstract 2

This study aimed to investigate the inhibitory mechanism of metabolic uncoupler on biofilm formation through surface characteristics and thermodynamics analysis.

## Graphical abstract

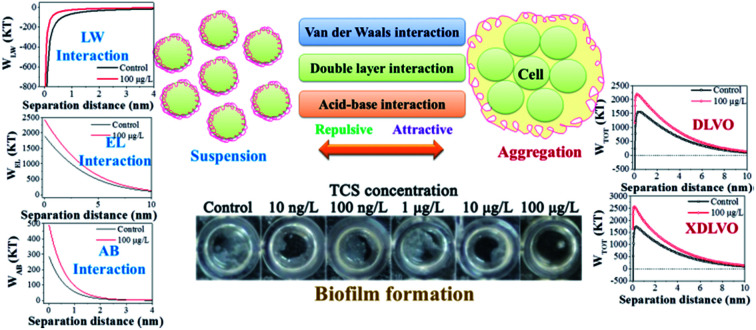

## 1. Introduction

Membrane separation technology has been widely implemented over the last three decades and become one of the most promising technologies in drinking water provision, wastewater treatment and desalination.^1–3^ Although it has numerous advantages (high-quality effluent, automatic operation, easy scale-up and low space requirement) over conventional water treatment process,^4,5^ the major drawback and limitation encountered in the application of membrane separation system is membrane fouling which can be classified into four types namely particulate, inorganic, organic and biofouling based on its composition.^6–9^ While other types of fouling can be controlled by pre-treatment, biofouling represents the Achilles heel in membrane filtration systems because microorganisms are ubiquitous in any feed water system.^8,9^ Biofouling refers to the undesirable accumulation of microorganism defined as biofilm on a membrane surface that leads to increased transmembrane pressure and energy consumption, as well as decreased permeate water flux.^10–12^ Although traditional biofouling control methods, including hydraulic cleaning,^13,14^ acid–alkali treatment^15–17^ or common oxidants treatment such as chlorine and hydrogen peroxide,^16–19^ have been applied for alleviating the biofouled membrane, few of these methods could effectively control the membrane biofouling rather than compromising the adverse effect on membrane lifespan and performance.^10^

Numerous biological methods for biofouling control and inhibiting biofilm formation have recently gained increasing attentions and introductions.^19^ Compared with the traditional biofouling control angles which aim to remove or kill microorganisms, biological control methods include inhibiting microbial attachment, interfering biofilm formation and increasing biofilm dispersal mainly through quorum sensing (QS) system inhibition, EPS hydrolysis and energy uncoupling.^20^ In the field of wastewater treatment, energy uncoupling means the tight coupling between catabolism and anabolism, which is disassociated without affecting the substrate utilization rate.^21,22^ The investigation of metabolic uncoupler mainly focused on the field of excess sludge reduction^23^ and bacteria activity,^24^ which can effectively induce the occurrence of energy uncoupling and the reduction of ATP due to the reduction of proton motive force on two sides of the cytoplasmic membrane.^25–28^ It is attractive that metabolic uncouplers were found to promote different-age biofilms detachment, inhibit microorganism attachment and suppress the production of autoinducer-2 (AI-2), a common QS signal used in interspecies cellular communication during biofilm formation.^29–31^ In addition, the addition of a metabolic uncoupler, 4-nitrophenol (4NP), was also reported to alleviate biofouling and reduce biofilm formation in membrane bioreactor (MBR) system.^32^ Considering several metabolic uncouplers are environmentally-benign and potential to application,^27,33^ the utilization of metabolic uncoupler presents a potential economic and high-efficient approach to mitigate biofilm formation in membrane separation system. Although some previous studies found the presence of uncoupler inhibited the production of ATP and QS signals which might be the cause of biofilm reduction,^29–31,34^ the potential effect of TCS on surface characteristics and thermodynamics properties would be important to understand the inhibitory mechanism of uncoupler on biofilm formation.

Most bacterial surfaces are negatively charged and contain hydrophobic surface components.^35^ In biofilm formation process, the initial colonist bacteria adhere to the surface through weak, reversible adhesion *via* van der Waals forces and hydrophobic effects.^36^ Hydrophobicity and hydrophilicity can directly affect the behaviour of bacteria to aggregate.^37^ Therefore, the aggregation and biofilm formation of bacteria are closely related to bacterial surface characteristics including the surface charge and hydrophobicity/hydrophilicity.^38,39^ Therefore, investigating the changes of bacterial hydrophobicity/hydrophilicity and surface charge induced by uncoupler would be beneficial for the understanding the inhibitory function of metabolic uncoupler.

The classical DLVO theory, which includes two types of interactions, namely van der Waals (LW) and electrostatic double layer interactions (EL), is often proposed to describe the stability of colloidal suspensions and colloidal membrane fouling in the field of colloid chemistry.^37,38^ Liu *et al.*^37^ applied the DLVO theory to investigate the flocculation characteristics and suspension stability of H_2_-producing photosynthetic bacteria, *Rhodopseudomonas acidophila*. Compared with the traditional DLVO theory, the XDLVO model additionally considers an acid–base (AB) (electro donor/electron acceptor) interaction between two surfaces immersed in a polar solvent.^38^ Surface thermodynamics and interfacial tension for many polymeric surfaces and microorganisms submerged in water have implied the AB interaction contribution.^40–42^ In the study of Xiao *et al.*,^43^ the XDLVO theory was explored to describe the combined effect of membrane characteristic and foulants (dextran, bovine serum albumin and humic acid) on hydrophobicity and surface charge in microfiltration process. Meinders *et al.*^44^ investigated the deposition efficiencies and reversibility of bacterial adhesion on various substratum surfaces and then found bacterial adhesion to the surface was more accurately described by the XDLVO approach. In the study of Kumar and Ting,^45^ they observed the presence of streptomycin increased biofilm formation by *Staphylococcus aureus* and *Pseudomonas aeruginosa* due to the changes in cell surface characteristics. Therefore, the microbial surface thermodynamic theories based on DLVO and XDLVO are the fundamental and accurate approaches to analyse and predict biofilm formation and aggregation.^46^

Herein, this study systematically analysed and evaluated the effect of a typical uncoupler, TCS, on biofilm formation, aggregation, flocculability, surface characteristics and thermodynamic properties of a Gram-positive bacteria, *Bacillus subtilis*, which has been widely studied as a representative bacterium for biofilm formation.^47–49^ We investigated the inhibitory effectiveness of TCS on biofilm formation in a wide concentration range and found the effective concentration was as low as 100 μg L^−1^. The bacterial surface characteristics and thermodynamic properties were evaluated by DLVO and XDLVO theories to elucidate the variation of bacterial surface interaction energy induced by TCS. This study aimed to investigate the potential inhibitory mechanism of metabolic uncoupler on biofilm and biofloc through surface characteristics and thermodynamic analysis. The results in this investigation might be helpful to further understand the relationship between bacterial surface characteristics and biofilm formation, which can promote the development of biofouling control approach based on metabolic uncoupler.

## 2. Materials and methods

### 2.1 Microbial strain and growth conditions

The Gram-positive bacteria strain *B. subtilis* (ATCC6051) was used in this study. *B. subtilis* is a common typical bacterium for biofilm studies.^48,49^ TCS (99%) was purchased from Acros Organics (Belgium). *B. subtilis* first cultivated in Tryptic Soy Agar (TSA) media in Petri dishes, stored at 4 °C and re-inoculated every week. Before each experiment, a 15 ml centrifuge tube containing 6 ml TSB (tryptic soy broth) liquid medium was inoculated with cell from the stock plate, and incubated at 37 °C for 24 h to late exponential phase until OD_595_ = 0.8 (∼10^8^ CFU ml^−1^). Prior to use, the cultivated bacteria in TSB media were centrifuged and washed twice with PBS buffer. The accurate bacteria concentration was measured by OD_595_ and plate counting using TSA plates. All other chemicals used were analytical grade, and solutions were prepared with ultrapure water (18.0 Ω, Pall, Cascada LS, USA).

### 2.2 Biofilm development

Biofilm cultivation conditions were aimed to produce microcolony or biofilm of *B. subtilis* in order to control difference induced by sample handing. In this study, the microbial biofilm were cultivated in TSB media in polystyrene 96-well plates (Costar, Coring, NY, USA). Microtiter wells were seeded with 200 μl bacterial suspension with an initial concentration of 2 × 10^6^ CFU ml^−1^. One 96 well plate, columns 1 to 3 were applied as blank (TSB media only), columns 3 to 4 were used as the control (without TCS addition) and columns 5 to 10 were applied as experimental group (with different TCS concentrations). The composition in each well is shown in ESI.† Microtiter plate was then incubated at 37 °C with shaking at 150 rpm in an incubator. After 24 h incubation, the liquid in each well was discarded by inverting the plate upside down to dump the cell suspension and the plate was washed by PBS buffer to remove loose cells. Then, the microtiter plate was placed in incubator at 60 °C for 30 min to fix the remaining biofilm in each well. Each well was added by 40 μl crystal violet (1%), and submerged for 20 min in order to stain the biofilm. After discarding the additional Crystal Violet, each well was washed twice by 200 μl sterilized DI water and then 200 μl 95% ethanol was added into each well to extract the Cry violet. The plate was eluted at 37 °C with shaking at 150 rpm in an incubator for 3 h, and then 50 μl of extracted ethanol in each well was transferred to a new 96-well plate and diluted 4 times by 95% ethanol to measure the absorbance of crystal violet at 595 nm using a microplate reader. All biofilm samples were prepared in sextuplicate in one column and absorbance measurements for each plate were run in parallel.

### 2.3 Bacterial particle size measurement and flocculation test

In this study, 50 ml centrifuge tubes were seeded with 20 ml TSB medium with an initial bacterial concentration of 2 × 10^6^ CFU ml^−1^. These tubes with or without TCS were then incubated at 37 °C with shaking at 150 rpm. After 24 h incubation, particle size distributions of the bacterial suspension in each tube were obtained using Mastersizer 2000 (Malvern Co., UK). *d* (0.5) is the median diameter. All cell samples were prepared in triplicate and particle size measurements for each sample were run in triplicate.

In the test of flocculation, *B. subtilis* cells with or without TCS were incubated for 24 h at 37 °C with shaking at 150 rpm. Then the *B. subtilis* treated by centrifugation at 12 000 rpm for 10 min and washed twice by 0.1 M NaCl solution. After discarding supernatant, cell pellets were re-suspended in NaCl solution with different concentrations and the absorbance of cell suspension was measured at 650 nm (*A*_0_). The rest of cell suspensions were centrifuged at 1000 rpm for 2 min, and then measured the optical density at 650 nm (*A*_t_). All cell samples were prepared in parallel and OD_650_ measurements for each sample were run in triplicate. The calculation formula of *F* can be written as:^37^1
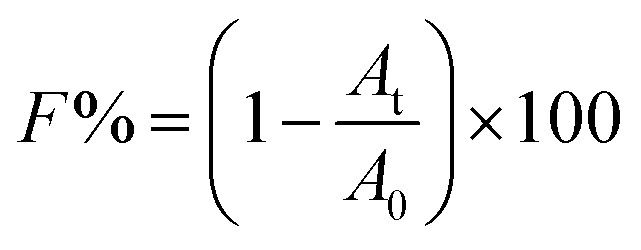


### 2.4 Zeta potential and contact angle measurement

*B. subtilis* cells in NaCl solution with gradient concentrations were harvested by the same method of flocculation test. The zeta potential of each sample was measured by a Zetasizer Nano ZS Instrument (Malvern Co., UK) at room temperature. After 24 h incubation of *B. subtilis* cells with or without TCS at 37 °C with shaking at 150 rpm. All cell samples in this portion were prepared in parallel and zeta potential measurements for each sample were in triplicate.

In the process of contact angle measurement, *B. subtilis* cells were first incubated with or without TCS for 24 h, and then homogeneous cellular layers were gained on 0.45 μm acetate cellulose membranes by suction filtration. These samples were washed twice by DI water and then placed into Petri dishes for 12 h in dehydrator. The measurements of contact angles were used by sessile drop approach with a drop of ultrapure water, formamide, and 1-bromonaphthalene. All cellular layer samples were prepared in parallel and contact angle values for each sample were based on arithmetic means of at least ten independent measurements.

### 2.5 Surface thermodynamics analysis by DLVO and XDLVO

In this study, the DLVO theory indicates the total interaction energy between two colloids as the sum energy of van der Waals (LW) and electrostatic (EL) interaction.

2 *W*^DLVO^_TOT_ = *W*^LW^ + *W*^EL^

where *W*^DLVO^_TOT_ is the total interaction energy between two colloids in liquid predicted by DLVO theory, *W*^LW^ is the LW interaction term and *W*^EL^ is the EL interaction term.

Later, van Oss^50^ suggested that the energy balances performed for aqueous systems must account for the acid and base interaction energy in addition to the LW and EL interaction energy. Considering the AB interaction energy, the XDLVO approach can be written as

3 *W*^E−DLVO^_TOT_ = *W*^LW^ + *W*^EL^ + *W*^AB^

where *W*^E−DLVO^_TOT_ is the total interaction energy between two bacterial cells immersed in water predicted by XDLVO approach and *W*^AB^ is the AB interaction term.

The detailed calculations for the terms in the DLVO and XDLVO theories are shown in ESI.†

### 2.6 EPS extraction and measurement

In this study, 50 ml centrifuge tube was seeded with 20 ml TSB medium with an initial bacterial concentration of 2 × 10^6^ CFU ml^−1^. These tubes with or without TCS were then incubated at 37 °C with shaking at 150 rpm. After 24 h incubation, 20 ml of bacterial liquid media was centrifuged at 8000*g* for 15 min at 4 °C. Cell pellet left in the centrifuge tube was re-suspended to its original volume by NaCl solution (0.05% w/w). The cell suspension was placed in a water bath at 60 °C for 30 min, and then centrifuged at 8000*g* for 15 min at 4 °C. The organic matter in the supernatant was regard as EPS. The analysis of EPS by chemical methods mainly measured the contents of proteins and polysaccharides which were considered as the dominant components of EPS. The measurement of proteins was measured by the modified BCA assay and the concentration of polysaccharides was evaluated by phenol-sulfate acid method. All EPS samples were prepared in parallel and measurements of EPS samples were conducted in triplicate and the mean value was presented.

### 2.7 Data and statistical analysis

Means and standard errors for all experiments were calculated at each test point using Microsoft Excel. Data shown in the graphics was analysed by independent sample *t* tests with a significance level of 95% (*i.e.*, *p* < 0.05) showed by *.

## 3. Results

### 3.1 Inhibition of TCS on biofilm formation

**Fig. 1:**
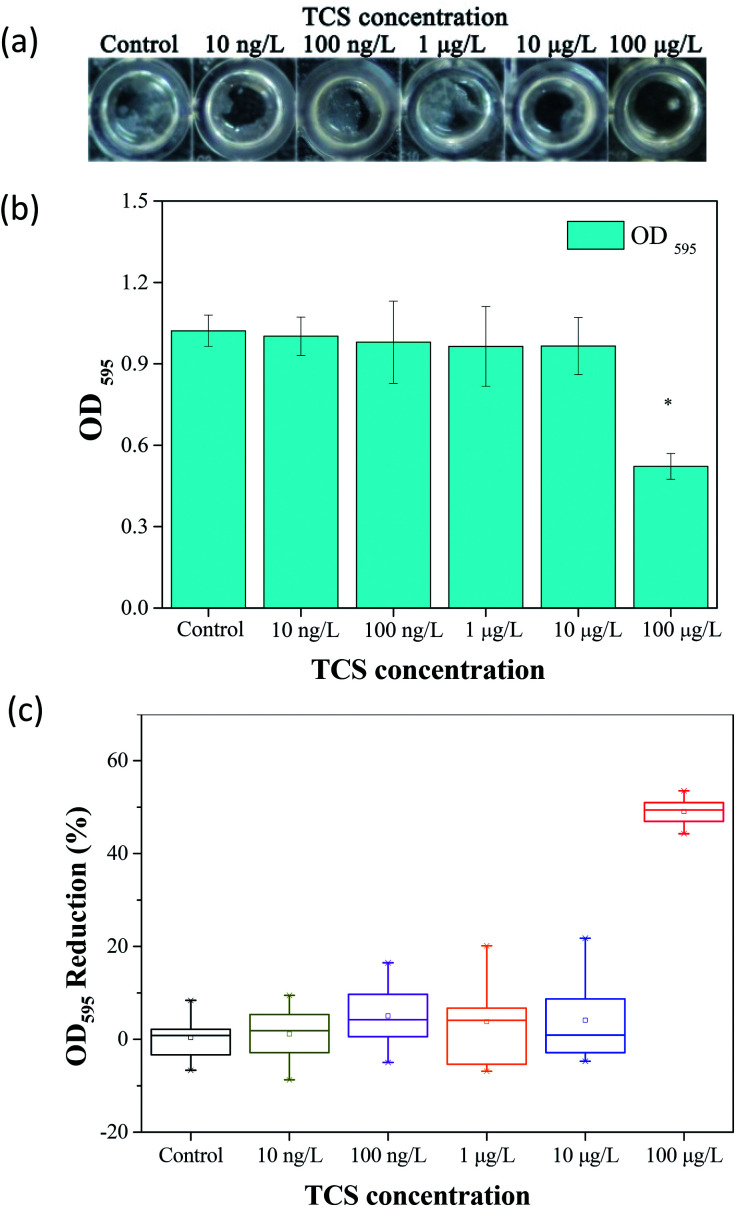
(a) Biofilm formation of *B. subtilis* in different TCS concentrations. (b) Shown on the OD_595_ in different incubation conditions. (c) Biofilm formation of *B. subtilis* cells were significantly inhibited by 100 μg L^−1^ of TCS. Asterisk indicates significant difference compared to control sample (*p* < 0.05).

The effect of TCS on bacterial biofilm formation was studied in a wide concentration range using a Gram-positive bacterium, *B. subtilis*. The schematic of biofilm development shown in Fig. S1.†Fig. 1(a) showed the formed biofilm after 24 h incubation under different TCS concentration. Visible rough biofilms or microcolonies were found in the bottom of 96 well plates in the control group and the similar biofilms also were observed after 24 h incubation in test groups with TCS concentration from 10 ng L^−1^ to 10 μg L^−1^. In contrast, few biofilm was observed in the test group with TCS concentration at 100 μg L^−1^. Crystal violet approach was applied to quantify the formed biofilm in different TCS concentration and the results are shown in Fig. 1(b). The OD_595_ values of control samples and test samples with TCS concentration from 10 ng L^−1^ to 10 μg L^−1^ were around 1.0. However, after 24 h incubation and stained by crystal violet, the OD_595_ decreased to only about 0.5 in the wells with TCS (100 μg L^−1^). The inhibition effectiveness of TCS on biofilm formation by 24 h incubation is shown in Fig. 1(c). The presence of TCS lower than or equal to 10 μg L^−1^ did not induce significant reduction of *B. subtilis* biofilm. When TCS concentration increased from 10 μg L^−1^ to 100 μg L^−1^, the biofilm formation was significantly reduced by over 50%. In fact, there are a great number of attached cells at the bottom of a well could not be directly observed by eyes, but these cells could be stained by crystal violet. In addition, it was observed that TCS at 100 μg L^−1^ did not significantly impair the growth of *B. subtilis* (data shown in Fig. S2†). In previous study, it was found the presence of TCS at higher concentration (4 mg L^−1^) obviously inhibited the formation of aerobic granular.^34^ However, it was also reported that TCS over 1 mg L^−1^ directly induced sludge reduction in activated sludge system.^23,33^ It is notable that in this study, we found that lower TCS concentration (100 μg L^−1^) inhibited the biofilm formation of *B. subtilis* without the inhibition of bacterial growth. In other words, the inhibitory function of TCS on biofilm formation at low concentration was not induced by the growth inhibition. This found implied the application of TCS at 100 μg L^−1^ for biofilm inhibition can be considered as a kind of biological biofouling control approach.

### 3.2 The variation of biofloc particle size induced by TCS addition

**Fig. 2:**
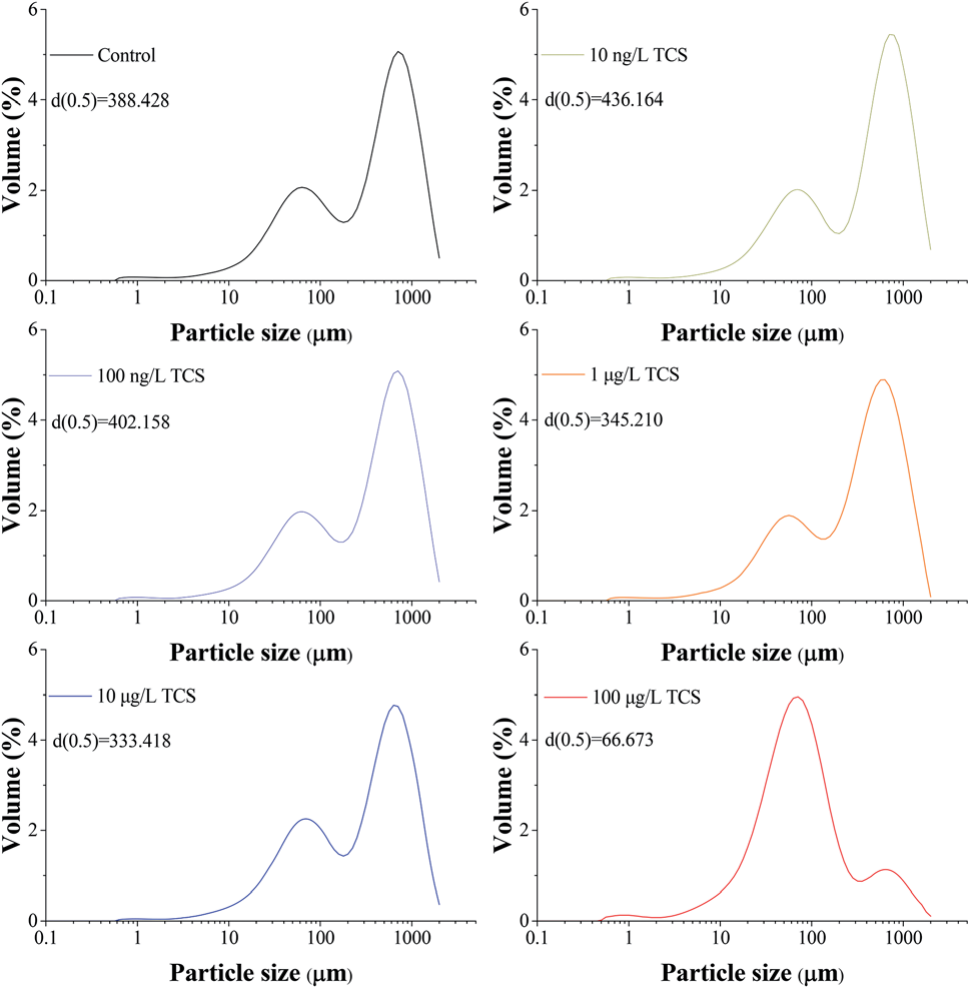
Particle size distributions of *B. subtilis* biofloc after 24 h incubation shaking at 150 rpm with or without TCS addition.

The particle size distributions of *B. subtilis* biofloc after 24 h incubation with or without TCS addition were shown in Fig. 2. Considering the bacterial biofloc regarded as a kind of biofilm without contact surface, the formation of biofloc and biofilm should have the similar mechanism. It was observed that the control sample has two intensive particle size distribution areas which concentrated on 60 and 700 μm and the volume percent of particle size distribution area on 700 μm was obviously higher than that on 60 μm. However, the volume percent of 60 μm was about fourfold to that of 700 μm in 100 μg L^−1^ TCS sample, indicating that the formation of biofloc and cells aggregation was substantially inhibited by 100 μg L^−1^ TCS. This result suggested that the bacteria aggregation was repressed or the biofloc tended to disperse when bacteria incubated with TCS at 100 μg L^−1^. In addition, the value of *d* (0.5) in test sample with 100 μg L^−1^ TCS was apparently lower than that of control, which also suggested the formation of *B. subtilis* biofloc was affected by TCS at 100 μg L^−1^. Meanwhile, the particle size distribution of other test groups were similar with that of control.

### 3.3 Effect of TCS on EPS secretion

**Fig. 3:**
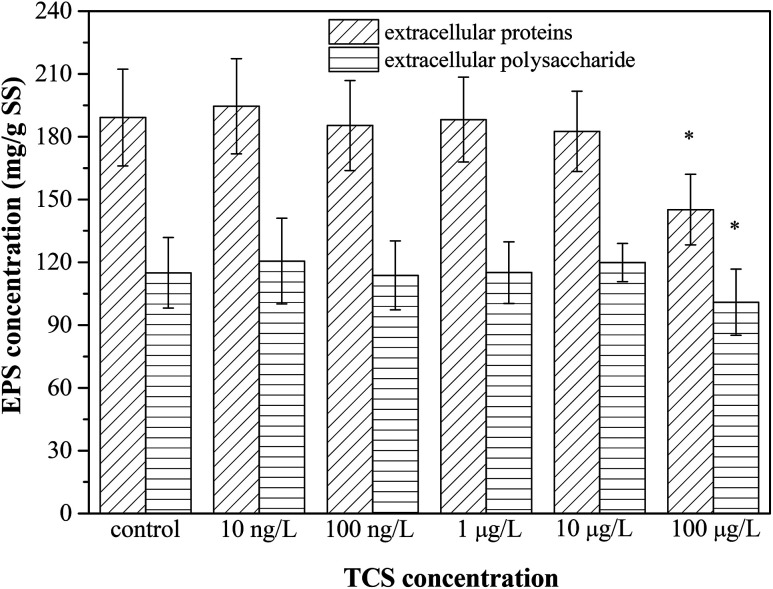
The effect of TCS on extracellular proteins and polysaccharides production per unit biomass after 24 h incubation.

Give the importance of EPS in the process of biofloc and biofilm formation,^39,51^Fig. 3 presents the contents of extracellular proteins and polysaccharides in EPS extracted from *B. subtilis* cells incubated with or without TCS after 24 h. The concentrations of polysaccharides and proteins in EPS were not affected by TCS at the concentration from 10 ng L^−1^ to 10 μg L^−1^, implying the secretion of EPS was not affected by TCS at these concentrations. However, the apparent reduction in the contents of proteins and polysaccharides in EPS extracted from *B. subtilis* cells after 24 h exposure to 100 μg L^−1^ TCS was observed in Fig. 3, suggesting the secretion of EPS was inhibited by 100 μg L^−1^ TCS which contributed to the reduction of biofilm and biofloc particle size. Some previous studies^23,33^ reported that dosing 1 mg L^−1^ TCS in activated sludge inhibited the normal growth of sludge and increased the secretion of EPS and soluble microbial products (SMP) which might be because the defence mechanism were stimulated by the presence of TCS. However, this study found that the presence of TCS at 100 μg L^−1^ did not inhibit the growth of *B. subtilis* and only control the process of biofilm formation, implying the defence mechanism was not motivated. Jiang and Liu^34^ suggested that under energy deprivation condition, microorganisms would first satisfy their basic metabolic requirements compared to synthesis of “luxury” macromolecules, such as EPS. Therefore, TCS at different concentrations may induce different effects on microbial EPS production.

### 3.4 Effect of TCS on bacterial zeta potential and flocculability

**Fig. 4:**
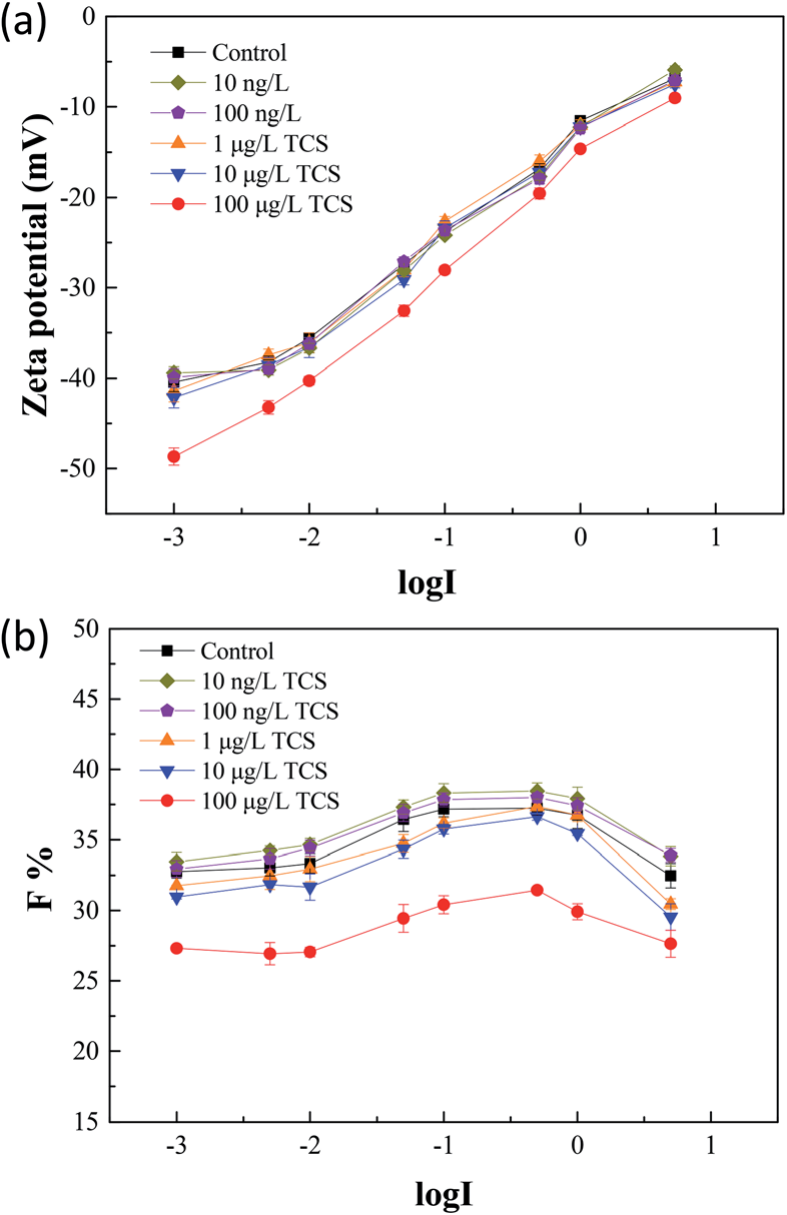
(a) After 24 h incubation of *B. subtilis* with or without TCS, zeta potentials were measured in bacteria re-suspended solution with gradient concentration of NaCl; (b) the *F* values of flocculability in control and test groups with TCS addition were calculated by eqn (1).

Zeta potential is an important parameter which characterizes the physicochemical properties of bacterial cell surface and has a tight relationship with bacterial aggregation and disaggregation processes.^52^ After 24 h incubation of *B. subtilis* with or without TCS, the values of zeta potential in bacterial re-suspended solution with gradient concentration of NaCl were shown in Fig. 4(a). The zeta potentials in the control group and the test groups reduced negative charge with the increasing electrolyte concentration due to the double layer compression. In the control group, the zeta potential increased from −40.43 to −6.85 mV with an increase of NaCl concentration from 0.001 to 5 M. At the same time, the variations of test groups with TCS concentration from 10 ng L^−1^ to 10 μg L^−1^ were similar with control. However, the zeta potential in test group (100 μg L^−1^ TCS) increased from −48.66 to −9.03 mV with the same NaCl concentration variation. It was observed that the zeta potential at each point of NaCl concentration in the test group (100 μg L^−1^ TCS) was more negative than that of the control group. This finding indicated that the presence of 100 μg L^−1^ TCS in bacterial growth phase induced the changes of bacterial surface charge. The more negative surface charge might be induced by the changes of EPS secretion.^39^ In addition, the difference of zeta potential between control sample and 100 μg L^−1^ TCS sample decreased with an increase in ionic strength. Generally, the bacteria with more negative charge is expected for lower membrane fouling tendency in membrane filtration system.^32^ The zeta potential of *B. subtilis* after 24 h incubation with 100 μg L^−1^ TCS was more negative than that of control, suggesting that the presence of 100 μg L^−1^ TCS during the process of cell culture enhanced the cell surface negative charge. This result could be an important cause for the reduction of the biofloc particle size and biofilm in previous sections.

Fig. 4(b) showed the flocculability of *B. subtilis* re-suspended in different NaCl concentration solution. A higher *F* value indicates a less stable cell suspension and also means a stronger flocculability of bacterial cells. The *F* values in the control group and test groups increased with the increasing electrolyte concentration from 0.001 to 0.1 M which implied the increase of ionic strength within limits could improve the flocculability of *B. subtilis*. As the result in zeta potential measurements, the zeta potential decreased with the increase of ionic strength and therefore, caused the reduced contribution of electrostatic repulsive interaction between two cells. However, the increasing tendencies of flocculability in the control group and test groups were both reversed when the NaCl concentration over 0.1 M. This contradictory phenomenon should be due to the occurrence of “hydrophobization” and “salting out” in high salt solution.^53^ Notably, the flocculability of *B. subtilis* incubated with 100 μg L^−1^ TCS was evidently worse than that of control and other test groups. The reduction of flocculability was consisted with the difference of zeta potential, indicating the weaken flocculability of *B. subtilis* incubated with TCS (100 μg L^−1^) was attributed to its more negative surface charge in low ionic strength solution. However, the difference of flocculability between the control and TCS sample (100 μg L^−1^) was reduced due to the NaCl concentration over 0.1 M. This variation should be induced by the lower contribution of the double layer interaction and dehydration at higher ionic strength.

### 3.5 Effect of TCS on *B. subtilis* cells surface characteristics

The surface characteristics of bacteria such as contact angle and surface charge have significant effect on bacterial hydrophobicity and flocculability.^37,46^ As shown in Table 1, contact angles were measured for *B. subtilis* which were incubated with or without TCS. Generally, contact angle divided into two categories: *θ*_w_ < 90° is wetting and *θ*_w_ > 90° is non-wetting. It is important to indicate that the hydrophobic interactions between two surfaces become effective at *θ*_w_ > 65° and the hydrophilic interactions at *θ*_w_ < 65°.^54^ In this study, the contact angles of control group and test groups were all below 65°, suggesting that all *B. subtilis* surfaces were hydrophilic and hydrophilic interactions were dominant between *B. subtilis* cells and water. However, the contact angle of *B. subtilis* in the control group and cells incubated with TCS (100 μg L^−1^) were obviously different, implying the presence of TCS (100 μg L^−1^) could increase the hydrophilicity of *B. subtilis* cells. Xie *et al.*^55^ reported that the increase of water contact angle was favourable for the formation of biofloc. Therefore, the decreased water contact angle observed in 100 μg L^−1^ TCS sample would be unbeneficial for the formation of biofloc and biofilm. Moreover, the contact angles of test groups with TCS from 10 ng L^−1^ to 10 μg L^−1^ were not significantly changed.

**Table 1:** Average contact angle of bacterium cultured in control and TCS condition

TCS concentration	Contact angle *θ* (°)		
	Water	1-Bromonaphthalene	Formamide
Control	34.0 ± 0.9	47.0 ± 1.4	32.5 ± 1.6
10 ng L^−1^ TCS	35.1 ± 1.1	45.9 ± 1.2	31.6 ± 1.3
100 ng L^−1^ TCS	34.7 ± 1.2	46.4 ± 1.3	32.9 ± 1.4
1 μg L^−1^ TCS	33.6 ± 1.3	48.1 ± 1.1	34.2 ± 1.2
10 μg L^−1^ TCS	33.4 ± 1.1	48.9 ± 0.9	33.1 ± 1.3
100 μg L^−1^ TCS	28.4 ± 1.2	54.9 ± 1.3	41.2 ± 1.1

Based on contact angle measurements, surface tension parameters and interfacial free energy of *B. subtilis cells* cultured with or without 100 μg L^−1^ TCS were calculated and showed in Table 2. The data in Table 2 showed both control sample and test sample (100 μg L^−1^) had high electron donor components (*γ*_B_^−^) and relatively low electro acceptor components (*γ*_B_^+^), indicating the *B. subtilis* cells were typically characterized by a high electron donor monopolarity. The polar component (*γ*^AB^_B_) had a little decrease from control sample to test sample (100 μg L^−1^ TCS) indicating the AB interaction between two *B. subtilis* cells was affected by TCS presence. The interfacial free energy (Δ*G*_ad_) values in Table 2 provide a quantitative insight regarding the hydrophobicity/hydrophilicity of bacterial cells.^38^ Positive values of the interfacial free energy represent the hydrophilic surfaces, while negative values indicate the hydrophobic surfaces. The Δ*G*_ad_ of test sample (TCS at 100 μg L^−1^) was more positive than that of control sample, also suggesting that the bacterial surface of *B. subtilis* incubated with TCS (100 μg L^−1^) was more hydrophilicity. Generally, the cell surface with more hydrophobicity is more easily to form biofilm and biofouling on membrane surface^56^ and therefore, the effect of TCS on *B. subtilis* cells surficial hydrophobicity/hydrophilicity might play an important role in the process of biofilm inhibition.

**Table 2:** Surface energy parameters of *B. subtilis* cultured with or without TCS

TCS concentration	Surface energy (mJ m^−2^)					
	*γ* ^LW^_B_	*γ*_B_^+^	*γ*_B_^−^	*γ* ^AB^_B_	*γ*_BL_	Δ*G*_ad_
Control	31.04	1.80	44.36	17.88	−11.07	22.13
100 μg L^−1^ TCS	27.54	1.02	59.09	15.53	−20.97	41.94

### 3.6 Effect of TCS on cells interaction energy predicted by DLVO and XDLVO

The flocculation stability and aggregation of bacterial cells could be predicted from the interaction energy between bacterial cells.^55^ In the DLVO theory, the van der Waals attraction decreases as an inverse power of the distance between the particles, and the electrostatic repulsive energy is an approximately exponential function of the distance between the cell particles.^41^ The effects of 100 μg L^−1^ TCS on *W*_LW_ and *W*_EL_ in the prediction of DLVO are described in Fig. 4(a) and (b). Fig. 4(c) shows the DLVO interaction energy profiles of *B. subtilis* cells incubated with or without 100 μg L^−1^ TCS as a function of the separation distance between bacterial cells. As shown Fig. 4(c), the total interaction energy variation tendencies of *B. subtilis* cells incubated with or without TCS are similar at large separation distances (>8 nm). In two samples, at separation distance smaller than 8 nm, the EL repulsion dominated and the total interaction was repulsive. In the total interaction energy profiles, the existence of the primary energy maximum plays important role in determining the aggregation of bacterial cells.^56^ Bacterial cells can overcome the primary energy barrier and then fall into a deep primary energy minimum at very close contact and aggregate irreversibly.^57^ However, if bacteria cells cannot overcome the energy barrier, they would not aggregate with other cells. Therefore, a higher primary energy barrier implies a more stable bacterial suspension and more resisting aggregation and biofilm formation. The primary energy maximum of *B. subtilis* cells incubated as control (1564.29 kT) was evidently less than that of *B. subtilis* cells incubated with TCS at 100 μg L^−1^ (2194.07 kT). This variation indicated that when *B. subtilis* cells incubated with TCS (100 μg L^−1^), bacteria must consume more energy to overcome energy barrier to aggregate than the control cells. In this respect, the bacteria cells with higher primary energy barrier would be expected for poor flocculability and biofilm formation. The prediction of the control sample and test sample (100 μg L^−1^ TCS) by DLVO approach was consistent with the analysed results in biofilm, biofloc particle size and flocculability tests.

**Fig. 5:**
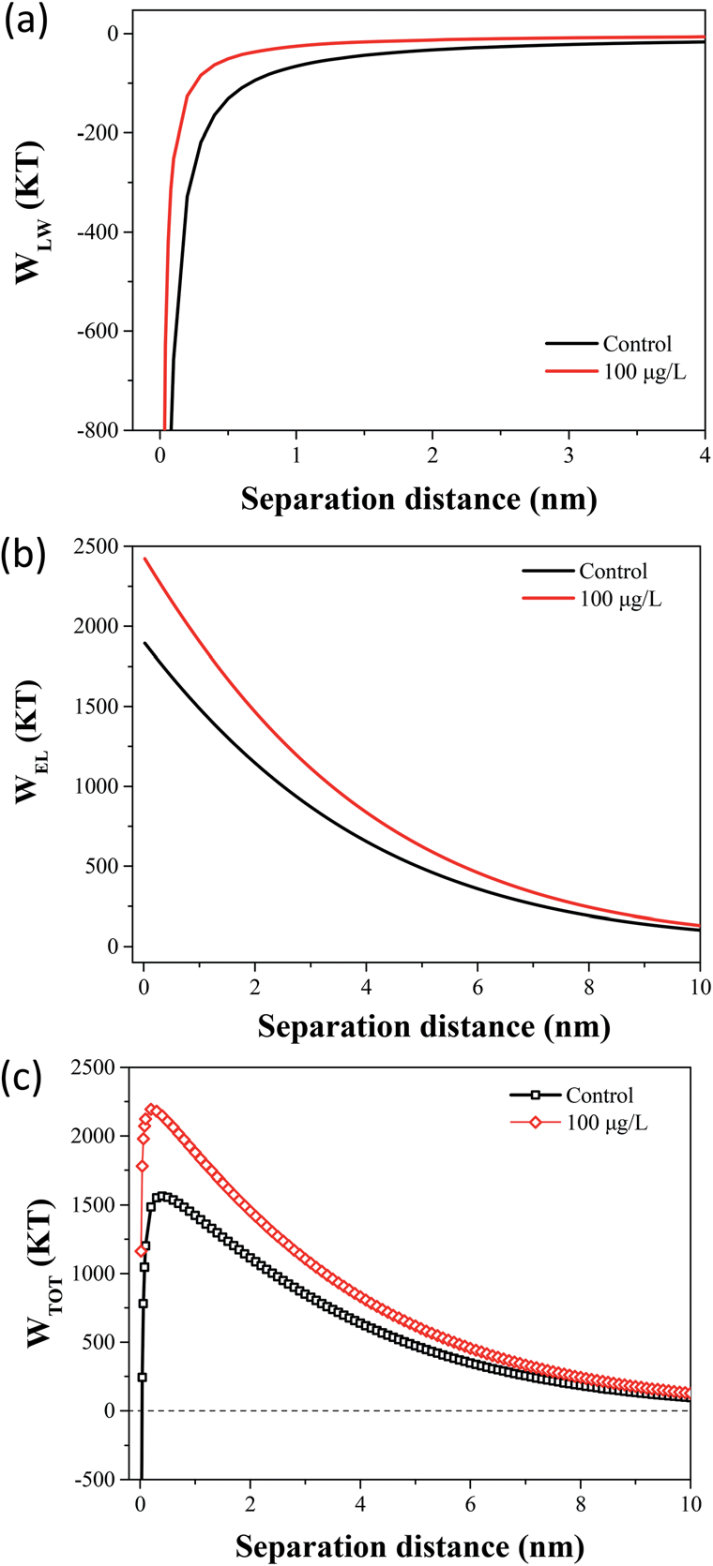
(a) The effect of 100 μg L^−1^ TCS on *W*_LW_ in the prediction of DLVO. (b) The effect of 100 μg L^−1^ TCS on *W*_EL_ in the prediction of DLVO. (c) Total interaction energy profile as a function of particle distance with the prediction of the classical DLVO theory.

**Fig. 6:**
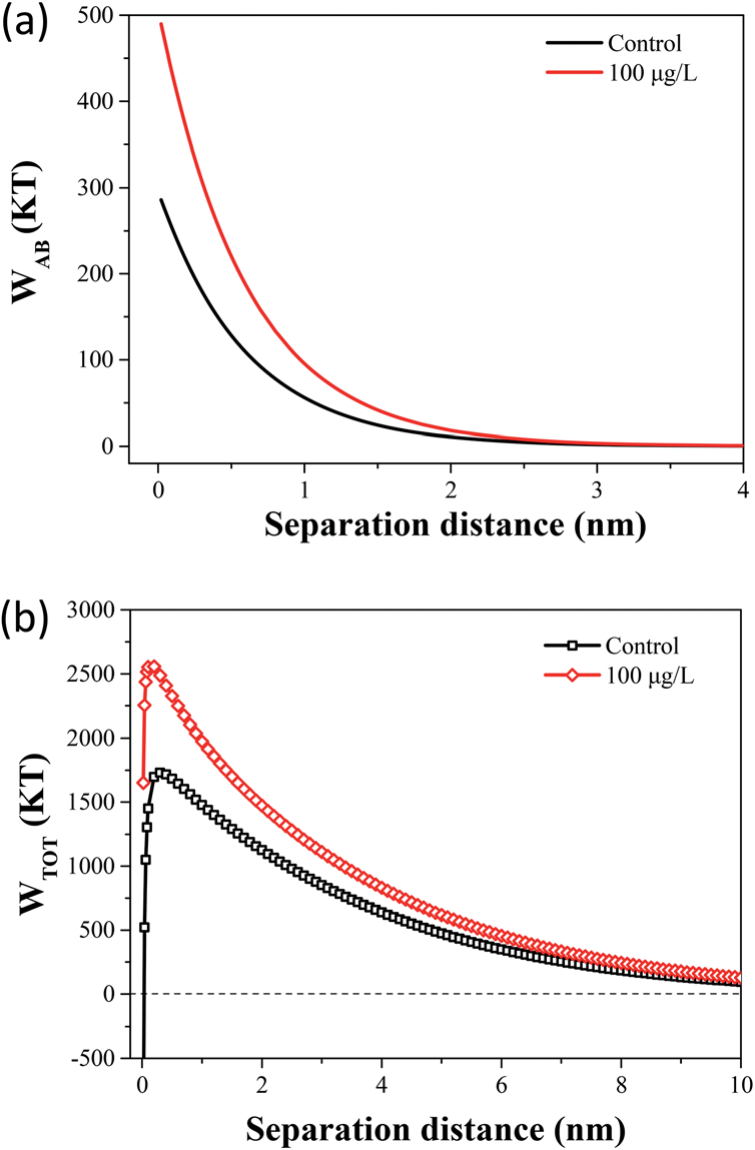
(a) The effect of 100 μg L^−1^ TCS on *W*_AB_ in the prediction of XDLVO. (b) Total interaction energy profile as a function of particle distance with the prediction of the XDLVO theory.

Compared with DLVO theory, the Lewis acid–base interaction in the XDLVO theory provides an additional asset to the increase or decrease in the total energy barrier.^45^ The effect of 100 μg L^−1^ TCS on *W*_AB_ in the prediction of XDLVO and the XDLVO interaction energy profiles of *B. subtilis* cells incubated with or without TCS as a function of the separation distance were shown in Fig. 5. For the control group and test group (100 μg L^−1^), the XDLVO approach predicted repulsion which was agreement with the DLVO theory prediction. However, the primary energy barriers in the control group and test group showed obvious increment due to the addition of AB interaction. The primary energy barrier of *B. subtilis* cells incubated with TCS at 100 μg L^−1^ (2557.04 kT) was significantly higher than that of the control (1731.99 kT) and the difference of barrier got larger compared with DLVO prediction. In XDLVO prediction, the increment of primary energy barrier induced by 100 μg L^−1^ TCS suggested that the bacterial cells incubated with TCS (100 μg L^−1^) would overcome higher primary energy barriers to aggregate or develop biofilm compared with the prediction of DLVO. In addition, compared with DLVO theory, the total interaction energy barrier increased apparently in the control and test group through XDLVO calculation, indicating that the AB interaction played an important role in *B. subtilis* cells interaction. This prediction by XDLVO theory indicated a better flocculability and biofilm formation would be observed in the control group compared to the test group (100 μg L^−1^ TCS) (Fig. 6).

## 4. Conclusions

Low-dose of TCS was found to effectively inhibit the biofilm formation and secretion of EPS by *B. subtilis*, suggesting it was perspective in the investigation for biofilm control. Compared to the control, smaller biofloc, poorer flocculability, more negative surface charge and higher hydrophilicity were observed in the test sample after treated with 100 μg L^−1^ TCS. This study indicated that *B. subtilis* cells incubated with TCS at optimal concentration need to consume more energy to overcome the primary energy barrier, so as to aggregate or develop biofilm as predicted by DLVO and XDLVO. The analysis of cell interaction energy and surficial characteristics induced by TCS addition provided deeper understanding of the biofilm control process and biofilm inhibitory mechanism based on metabolic uncoupler.

## Conflicts of interest

There are no conflicts to declare.

## Supplementary Material

RA-008-C8RA02315H-s001
