# Supplementary material for: Effect of metabolic uncoupler, 3,3′,4′,5-tetrachlorosalicylanilide (TCS) on Bacillus subtilis: biofilm formation, flocculability and surface characteristics
Source: RSC Adv. 2018 May 1;8(29):16178–86. doi: 10.1039/c8ra02315h (PMC9080271; doi:10.1039/c8ra02315h)

Electronic Supplementary Material (ESI) for RSC Advances.
This journal is © The Royal Society of Chemistry 2018

Supporting information

Effect of Metabolic Uncoupler, 3,3′,4′,5-tetrachlorosalicylanilide (TCS) on *Bacillus subtilis*: Biofilm Formation, Flocculability and Surface Characteristics

Xiao-Chi Feng, Wan-Qian Guo*[1], He-Shan Zheng, Qing-Lian Wu, Hai-Chao Luo, Nan-Qi Ren

State Key Laboratory of Urban Water Resource and Environment, Harbin Institute of Technology, Harbin 150090, PR China

State Key Laboratory of Urban Water Resource and Environment, Harbin Institute of Technology, Harbin 150090, PR China

Corresponding Author: Wanqian Guo, Address: *State Key Laboratory of Urban Water Resource and Environment, Harbin Institute of Technology, Harbin 150090, PR China, Email: guowanqian@126.com, Phone: +86-451-86283008.*

The detailed calculations in the DLVO and XDLVO theories

The surface energy parameters of bacteria cell which can be determined by the experiments of contact angle measurements. In the study of van Oss et al.,[1] the total surface tension of a pure substance are considered as the sum of LW and AB, yielding:

$$\gamma_i = \gamma_i^{LW} + \gamma_i^{AB} \tag{4}$$

Where γ_i is the total surface tension and γ_i^{LW} and γ_i^{AB} are the LW and AB components of surface tension, respectively. Hence, the total surface tension of bacterial cell and liquid in this work can respectively be expressed as:

$$\gamma_B = \gamma_B^{LW} + \gamma_B^{AB} \tag{5}$$

$$\gamma_L = \gamma_L^{LW} + \gamma_L^{AB} \tag{6}$$

The polar AB component of a substance's surface energy is given by:

$$\gamma_i^{AB} = 2\sqrt{\gamma_i^{+}\gamma_i^{-}} \tag{7}$$

Where γ_i^{+} is the electron acceptor parameter and γ_i^{-} is the electron donor parameter. The extended Young equation relates the contact angle of a liquid on a solid surface to the surface tension parameters of both the bacterial and the liquid is given by:[1, 3]

$$(1+\cos\theta)\ \gamma_L = 2\left(\left(\gamma_B^{LW} \times \gamma_L^{LW}\right)^{\frac{1}{2}} + \left(\gamma_B^{+} \times \gamma_L^{-}\right)^{\frac{1}{2}} + \left(\gamma_B^{-} \times \gamma_L^{+}\right)^{\frac{1}{2}}\right) \tag{8}$$

Where θ is the contact angle between the bacterial surface and the drop liquid. The surface energy components of bacterial surface can be calculated by measuring the contact angles using three different probe liquids and the surface tension properties (mJ/m^2) of probe liquids were showed in table 1.[3]

Table 1 Surface tension properties (mJ/m^2) of probe liquids at 20 °C

Liqud	γ_L	γ_L^{LW}	γ_L^{AB}	γ_L^{+}	γ_L^{-}
Water	72.8	21.8	51	25.5	25.5
1-bromonaphthalene	44.4	44.4	0	0	0
Formamide	58	39	19	2.3	39.6

The surface tension parameters for *B. subtilis* and drop liquid used in this study are given in:

$$\gamma_{BL} = \gamma_{BL}^{LW} + \gamma_{BL}^{AB} \tag{9}$$

Where

$$\gamma_{BL}^{LW} = \left(\sqrt{\gamma_B^{LW}} - \sqrt{\gamma_L^{LW}}\right)^2 \tag{10}$$

$$\gamma_{BL}^{AB} = 2\left(\sqrt{\gamma_B^{+}\gamma_B^{-}} + \sqrt{\gamma_L^{+}\gamma_L^{-}} - \sqrt{\gamma_B^{+}\gamma_L^{-}} - \sqrt{\gamma_B^{-}\gamma_L^{+}}\right) \tag{11}$$

With these equations, the interfacial free energy (ΔG_{ad}) between two bacterial particles and liquid could be calculated from Eq. (12)

$$\Delta G_{ad} = -2\gamma_{BL} = -2(\gamma_{BL}^{LW} + \gamma_{BL}^{AB}) \tag{12}$$

According to DLVO and XDLVO theories,[3-5] the expressions for LW, EL and AB interaction between two bacterial particle are given by Eq. (13) - (15), respectively.

$$W_{LW} = -\frac{A_{BLB}R}{12H} \tag{13}$$

$$W_{EL} = 2\pi\varepsilon R\psi_s^2 ln(1 + exp(-\kappa H)) \tag{14}$$

$$W_{AB} = \pi R\lambda\Delta G_{l_0}^{AB} exp\left(\frac{l_0 - H}{\lambda}\right) \tag{15}$$

Where H is the separation distance between the cells. *B. subtilis* cells are assumed to be spherical and R is the cell radius. ψ_s is the stern potential was replaced by zeta potential in this study. κ represents the inverse debye length which is related to the electric double layer interaction and can be calculated with Eq. (19). λ is the correlation length of molecules in the liquid medium. A is the haymaker constant which is an important parameter for

determining a polar free energy of interactions between two bacterial cells. The haymaker constant A is measured using the contact angle approach and can be estimated using Eq. (16) - (18):

$$A = 24\pi {l_0}^2 \gamma_i^{LW} \tag{16}$$

$$A_{iwi} = \left(\sqrt{A_{ii}} - \sqrt{A_{ww}}\right)^2 \tag{17}$$

$$A_{BLB} = \left(\sqrt{A_{BB}} - \sqrt{A_{LL}}\right)^2 = 24\pi {l_0}^2 \left(\sqrt{\gamma_B^{LW}} - \sqrt{\gamma_L^{LW}}\right)^2 \tag{18}$$

The γ_B^{LW} and γ_L^{LW} can be calculated from surface thermodynamic analysis. l_0 is the minimum equilibrium distance (≈0.157 nm)

$$\kappa = \left(\frac{e^2 \Sigma n_{0,i} z_i^2}{\varepsilon_r \varepsilon_0 kT}\right)^{\frac{1}{2}} \tag{19}$$

Where e is the electron charge, k is Boltzmann's constant, and T is absolute temperature. ε_0 is dielectric permittivity in the vacuum (=8.854×10^{-12} CV^{-1}m^{-1}), and ε_r is the relative permittivity of water (=79). $\Sigma n_{0,i}$ is the number concentration of ion i and z_i is valence of ion i. For this investigation, a background electrolyte of 0.01 M NaCl solution was assumed.

Reference

1. C. J. Van Oss, R. J. Good and M. K. Chaudhury, *Journal of Colloid and Interface Science*, 1986, **111**, 378-390.
2. J. A. Brant and A. E. Childress, *Journal of Membrane Science*, 2002, **203**, 257-273.
3. C. J. van Oss, *Colloids and Surfaces A: Physicochemical and Engineering Aspects*, 1993, **78**, 1-49.
4. X. M. Liu, G. P. Sheng, J. Wang and H. Q. Yu, *Applied microbiology and biotechnology*, 2008, **79**, 187-194.
5. X.-M. Liu, G.-P. Sheng and H.-Q. Yu, *Environmental science & technology*, 2007, **41**, 4620-4625.

Table S1.The composition in each well of biofilm test.

Column	Blank (volume fraction)	Control (volume fraction)	10 ng/L TCS (volume fraction)	100 ng/L TCS (volume fraction)	1 μg/L TCS (volume fraction)	10μg /L TCS (volume fraction)	100μg /L TCS (volume fraction)
Water	20%	10%					
TSB medium	80%	80%	80%	80%	80%	80%	80%
TCS solution			10% (100 ng/L TCS)	10% (1μg /L TCS)	10% (10μg /L TCS)	10% (100μg /L TCS)	10% (1 mg/L TCS)
Bacteria solution (2×10^7 CFU/mL)		10%	10%	10%	10%	10%	10%

Fig. S1 The schematic of biofilm development

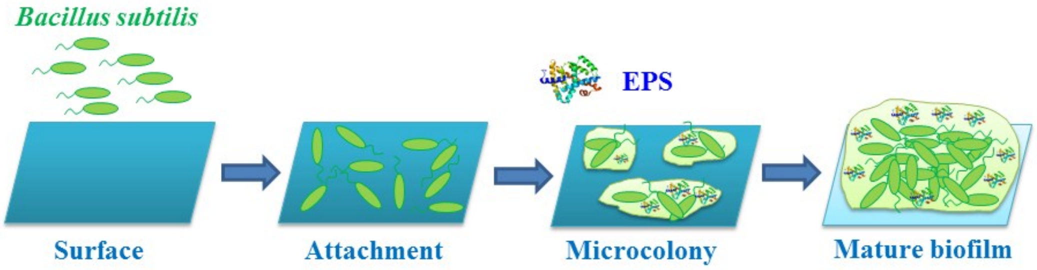

Fig. S2. The cellular biomass production by *B.subtilis* with different concentrations of TCS in 6 mL TSB media after 24 h incubation.

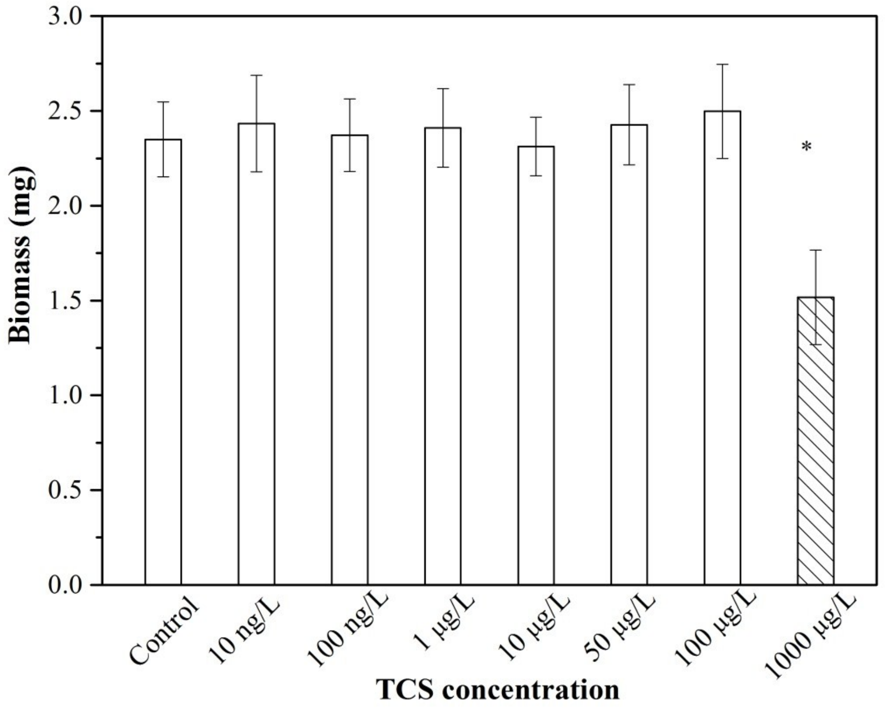

Supplement: RA-008-C8RA02315H-s001 [file RA-008-C8RA02315H-s001.pdf]
